# Hepatitis B Virus Infection in Patients with Blood Disorders: A Concise Review in Pediatric Study

**Published:** 2014-12-10

**Authors:** N Mansouri, A Movafagh, A Sayad, S Ghafouri-Fard, H Darvish, D Zare-Abdollahi, B Emamalizadeh, F Shahvaisizadeh, H Ghaedi, M Bastami, M Kayyal, M Hashemi, MH Heidari, A Nejatizadeh, M Zamani

**Affiliations:** 1 **Department of Medical Genetics, Shahid Beheshti University of Medical Sciences, Tehran, Iran.**; 2 **Department of Genetics, Islamic Azad University, Tehran, Iran.**; 3 **Department of Medical Anatomy, Shahid Beheshti University of Medical Sciences, Tehran, Iran.**; 4 **Department of Medical Genetics, Molecular Medicine Research Center, Hormozgan University of Medical Sciences, Bandar Abbas, Iran.**; 5 **Department of Neurogenetics, Iranian Center of Neurological Research, Tehran, Iran. **; 6 **Department of Medical Genetics, Faculty of Medicine, Tehran University of Medical Sciences, Tehran, Iran.**

**Keywords:** Malignancy, Heterogeneous population, HBV, HCV, Pediatric

## Abstract

Childhood Hepatitis B virus (HBV) infection causes both medical and public health challenges. Infants who acquire HBV parentally have up to 90% risk of developing chronic HBV infection. It is now estimated that approximately 10% of worldwide cancers are attributable to viral infection, with the vast majority (>85 %) occurring in the developing world. In this distribution, elevated rate and prevalence of HBV marker have been found in patients with malignancies as compared to the general population. By reviewing the web-based search for all Persian and English types of scientific peer review published articles initiated using Iran Medex, MEDLINE/PubMed, CINAHL and other pertinent references on websites about HBV and HCV blood disorders. The high prevalence of HBV and HCV infective markers was detected in patients with different malignancies. Moreover, identification of high prevalence of HBV infective markers in leukemia patients proposed strong association between hepatitis viral infections and leukemia.

## Introduction

Hepatitis B Virus (HBV) chronically infects over 350 million people and presents a global health threat [1]. Childhood HBV infection causes medical and public health challenges. Infants acquiring HBV parentally are about to developing chronic HBV infection up to 90%.

Infants acquiring HBV parentally are about to developing chronic HBV infection up to 90%.

It is now estimated that 10% of worldwide cancers is attributable to viral infection, with the vast majority (>85%) occurring in the developing countries [2]. Hepatitis viruses including HBV and HCV are present in large number of cancer cases such as Leukemias and they are responsible for the majority of hepatitis. Moreover, four DNA viruses namely Epstein Barr virus, certain human Papilloma virus subtypes, hepatitis B virus, and Kaposi sarcoma Herpesvirus (HHV-8) as well as two RNA viruses including adult T-cell leukemia virus (HTLV-1) are presently known to cause or contribute to tumor development. While HIV infection is not directly tumorigenic, HIV infection increases the incidence of certain tumors [2,6,7]. The aim of the present review was to investigate the prevalence and potential association between hematologic malignancies and Hepatitis B/C virus (HBV/HCV) infection in previous studies with heterogeneous population. Observing a new antigen in leukemia serum, there have been a number of reports on increasing prevalence of hepatitis B surface antigen (HBsAg) in the serum of patients with leukemia [8,9] especially acute myeloid leukemia (AML) and chronic myeloid leukemia (CML), [10-14].Being located in the Middle East, Iran has an intermediate-to-low prevalence of the HBV infection (2%). It is supposed that the HBV infection rate in young children diminishes significantly [15,16] after the implementation of the HBV National Vaccination Program (started in 1993). Following the prevalence of HBV throughout the world, patients co-infected with hepatitis B infection face the increased risk of leukemias. Some studies on hepatitis B virus infection and risk of lymphoma multicentre case-control study of lymphoma were conducted in six European countries. The results of aforementioned surveys add multiple myeloma to the list of potentially virus-associated lymphoma entities [17].In the following there is a number of researches into HBV in acute lymphoblasticleukemia one of which was done on Indian Patients [18]. High prevalence (3,932 cases) of hepatitis B and hepatitis C virus infections in Korean patients with hematopoietic malignancies was also reported [19]. Furthermore, preliminary data on the involvement of B, C and D hepatitis viruses in the etiopathogenesis of chronic lympho proliferative syndromes in Romania was documented [20]. From two separate reports in Russia [21,22], another incidence of hepatitis B virus infection in patients with blood disease was reported. In countries such as Turkey, high prevalence of chronic hepatitis B virus in children with cancer causes major problems in the management of cancer patients [6,23]. In South America, the very high prevalence rates for both HBV and HCV infection in patients with hematological-oncological disorders have been also documented in Nicaragua [24]. Another incidence and morbidity of 90 patients infected by hepatitis C virus in children with acute lymphoblastic leukemia that was reported is from Italian population [25]. In Far East, hepatitis B virus HBV, hepatitis C virus and HCV are introduced as the main causative agents of blood-born viral infection in Japan [26].Significantly increased the risk for HBV infection before vaccination where a diagnosis of leukemia was recorded at a teaching hospital in Baghdad [5]. Two cases of HBV reactivation in chronic myeloid leukemia patients were diagnosed before hepatitis B vaccination in Taiwan [4,11]. A total of 10,197 patients were diagnosed with chronic HBV infection in Sweden, five hundred sixty-seven of whom developed seven different cancers [27]. The results of European case-control study supported previous reports of an association between HBV infection with an elevated lymphoma risk as well as myeloma to the list of potentially virus-associated lymphoma entities [28].

## Material and Methods

The inclusion criteria for this review were that the documents were original, systemic review possessing qualitative and quantitative research, and published in English from January 1965 through December 2014 to assess existing knowledge on relationship between Hepatitis B virus infection and pediatric patients with blood disorder. In the search process for the literature the authors retrieved documents that contained malignancy, pediatric, HBV, HBC, virus-associated heterogeneous population, cancer sites for cancer registration, the most scientific databases were searched. English abstract, Russian abstract and full text of ProQues, MEDLINE/PubMed, CINAHL, (MeSH terms) were included. Articles that were not directly relevant to our specific objective questions were excluded. The software program as End Note was used to handle the proper references for instruction to author.

## Results

**Figure 1:**
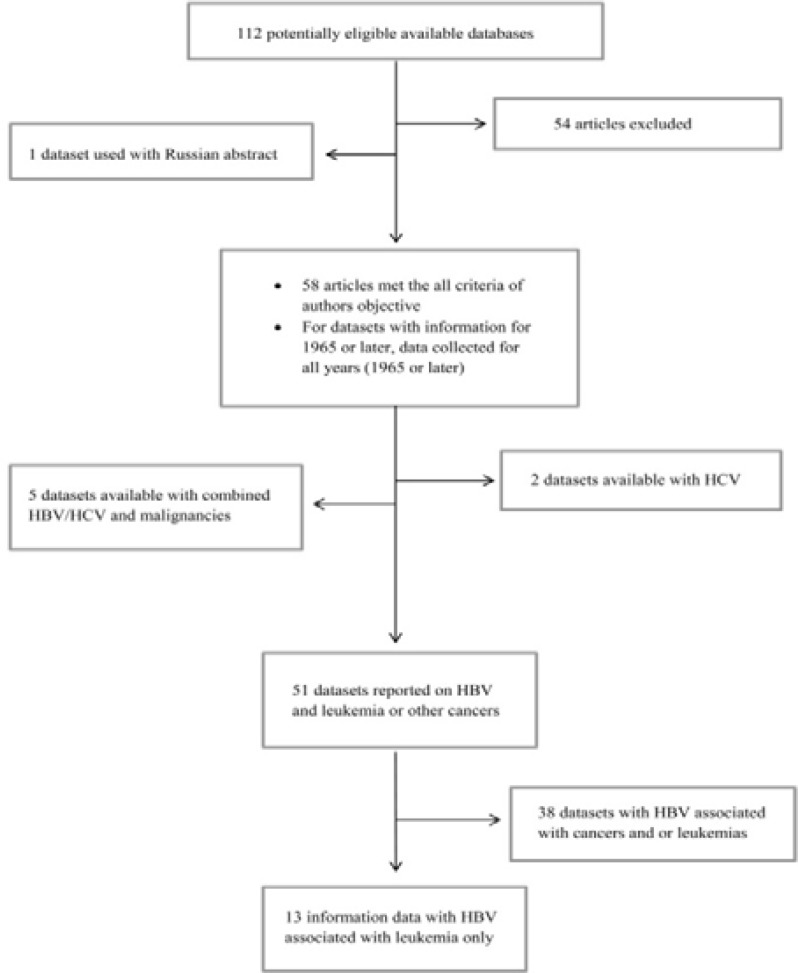
Database selection process

From the database, 112 articles were identified and after exclusion of duplicates, of which 58 article were original, systemic review with qualitative and quantitative research, and published in English met the criteria for inclusion in the present review article. Here, the major findings are summarized and presented under the following headings (Fig. 1).

**Clinical Manifestations of Hepatitis B Virus DNA**

**Figure 2:**
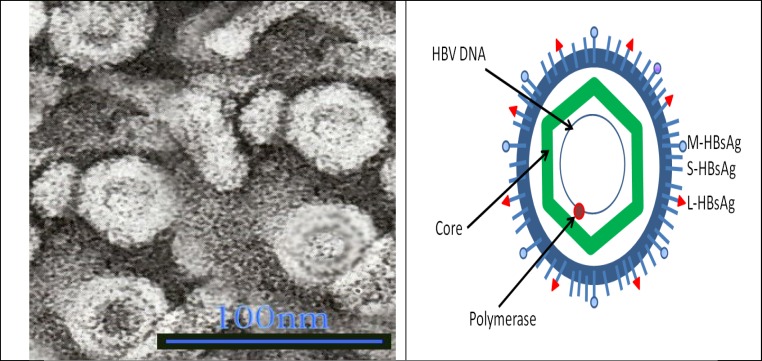
Hepatitis B Virus (HBV), the virus is a spherical particle with a diameter of 42nm (1nm = 0.000000001 metres) and is define as follows. Group: Group VII (ds-RT), Family, Hepadnaviridae, Genus: Orthhepadnavirus, Species: Hepatisis B virus.

HBV is a kind of the *hepadnaviridae* which includes members that can infect mammalian or avian species such as duck hepatitis B virus (DHBV). The *ca*. 3.2-kb genome of HBV is replicated by a virally encoded polymerase (HP), a specialized reverse transcriptase (RT). HP uses the viral pregenomic RNA (pgRNA) as the template to synthesize minus-strand viral DNA via its RNA-dependent DNA polymerization activity.  The minus-strand DNA is then used by HP as the template for plus-strand DNA synthesis. HP also has RNase H activity that is required to degrade the pgRNA template during minus-strandDNA synthesis. Minus-strand DNA synthesis is initiated by a novel protein priming mechanism in which HP itself serves a protein primer as well as the catalyst [29]. HBV enters the liver via the bloodstream, and replication occurs only in liver tissue. The intact, infectious virus is 42–47 nm in diameter and circulates in the blood in concentrations as high as 108 virions per ml. The inner core of the virus contains hepatitis B core antigen, hepatitis B antigen (HBeAg), a partially double-stranded 3,200-nucleotide DNA molecule, and DNA polymerase with reverse transcriptase activity. Hepatitis B surface antigen (HBsAg) is found both on the surface of the virus and as self-assembling, noninfectious spherical or tubular particles (Figure 2). The likelihood that newly infected persons will develop chronic HBV infection is dependent on their age at the time of infection. More than 90 percent of infected infants, 25–50 percent of children infected between 1 and 5 years of age, and 6–10 percent of acutely infected older children and adults develop chronic infection (i.e., they are HBsAg-positive but negative for immunoglobulin M antibodies to hepatitis B core antigen). Immuno suppressed persons (e.g., hemodialysis patients and persons with human immunodeficiency virus infection) are also at higher risk of developing chronic infection. Over 90 percent of perinatal HBV infections are asymptomatic, while the typical manifestations of acute hepatitis are noted in 5–15 percent of newly infected young children (1–5 years of age) and in 33–50 percent of older children, adolescents, and adults [30].

Diagnosis of acute or chronic hepatitis B virus (HBV) infection is based on the presence of HBV serologic markers such as hepatitis B surface antigen (HBsAg) and hepatitis B core IgM antibody (anti-HBcIgM), or the presence of HBV DNA detected by molecular assays. Although the diagnosis of acute and chronic HBV infection is usually made by serologic methods, detection and quantification of HBV DNA in serum are useful to: 

- Diagnose some cases of early acute HBV infection (before the appearance of HBsAg).
- Distinguish active from inactive HBV infection. 
- Monitor a patient's response to anti-HBV therapy. 

The presence of HBV DNA in serum is a reliable marker of active HBV replication. After 30 days following the infection, HBV DNA levels are detectable at its peak and gradually decrease and disappear when the infection resolves spontaneously. Since HBV DNA can be detected approximately 21 days before HBsAg, in cases of acute viral hepatitis with equivocal HBsAg test results, testing HBV DNA in serum may be a useful adjunct in the diagnosis of acute HBV infection, the quantification range of this assay is 20 to 170,000,000 IU/mL (1.30-8.23 log IU/mL). 

An "Undetected" result indicates that hepatitis B virus (HBV) DNA was not detected in the specimen. A "Detected" result with the comment, "HBV DNA level is <20 IU/mL (<1.30 log IU/mL). This assay cannot accurately quantify HBV DNA below this level" indicates that the HBV DNA level is below the lower limit of quantification for this study. When clinically indicated, follow-up testing with this assay is recommended in 1 to 2 months. 

A quantitative result expressed in IU/mL and log IU/mL indicates the degree of active HBV viral replication in the patient. Monitoring HBV DNA levels over time is important for assessing disease progression or monitoring a patient's response to anti-HBV therapy. 

A "Detected" result with the comment, "HBV DNA level is >170,000,000 IU/mL (>8.23 log IU/mL). This assay cannot accurately quantify HBV DNA above this level" indicates that the HBV DNA level is above the upper limit of quantification for this assay. 

An indeterminate result with the comment "Inconclusive Result: Submit a new specimen for testing if clinically indicated" indicates that inhibitory substances may be present in the specimen. When clinically indicated, collection and testing of a new specimen is recommended.

This assay was confirmed to have a lower limit of quantification (LLoQ) of 20 IU/mL based on probit analysis (95% hit rate), with good correlation to expected results and good linearity over the quantification range of the assay. Accuracy of results among hepatitis B virus (HBV) genotypes A to H was also confirmed. The mean difference in HBV viral load results between the VERSANT HBV DNA 3.0 Assay (bDNA) and the COBAS AmpliPrep/COBAS TaqMan HBV Test, v2.0 was 0.30 log IU/mL, with 96.1% (49 of 51 specimens with quantifiable results by both assays) of the differences falling within 0.85 log IU/mL of the mean difference and the individual differences ranging from 1.83 log IU/mL to -0.28 log IU/mL. A total of 25.5% (13 of 51 specimens) of the VL differences were >0.5 log IU/mL, and 5.9% (3 of 51 specimens) of the differences were >1.0 log IU/mL. No bias was observed over the range of HBV DNA levels tested. Similar viral load differences of >1.0 log IU/mL were observed in a published evaluation of this assay.

**Universal Childhood Prevalence and** **Prevention**

In 1992, the World Health Organization (WHO) set a goal for all countries to integrate hepatitis B vaccination into their universal childhood vaccination programs by 1997 [31]. Although, several countries have introduced hepatitis B vaccination for children into their national vaccination Programs.

HBV infection is vaccine-preventable disease in childhood. Nowadays the available recombinant hepatitis B vaccines are safe and immunogenic. The European Consensus Group on Hepatitis B Immunity has proposed a number of recommendations that are summarized in review section [32]. Hepatitis B vaccination has been shown to reduce the prevalence of chronic HBV infection and the incidence of HCC dramatically. In Turkey, HBV vaccination has been part of the routine childhood immunization schedule since 1998. In previous years, the prevalence of HBV infection among children was approximately 5% to 14% that was higher than the prevalence exhibited in other developing countries. In the studies performed during that period, the prevalence of HBV infection among children with cancer was reported to be as high as 20% to 65%. None of the patients had received childhood immunization, and the prevalence of HBV infection was 9.4% at the time of diagnosis [6,23] in a the previous study conducted in our clinic that examined the years between 1995 and 1998. Europe Bulgaria adopted the World Health Organization recommendation of routine universal infant vaccination against hepatitis B in 1991. 256 children in the hemato-oncology unit at the children's welfare teaching hospital, Baghdad, Iraq between September 2007 and June 2008 were studies [5] in order to investigate the incidence and role of hepatitis B virus (HBV) vaccination in preventing infection. In Taiwan, the prevalence of chronic infection in children declined by more than 90% [33]. In Gambia, the prevalence of chronic infection among children declined from 10.0% to 0.6% after implementation of universal infant hepatitis B vaccination. Similar declines in prevalence of chronic infection associated with infant and childhood hepatitis B vaccination have been demonstrated in China, Indonesia, Senegal, Thailand, and among Alaska Natives. Implementing universal infant hepatitis B vaccination in Taiwan, the incidence of HCC among children declined from 0.7 to 0.36 per 100,000 [34]. In the highly endemic countries in Asia where hepatitis B is the leading cause of chronic hepatitis, cirrhosis and hepatocellular carcinoma (HCC), the majority of infections are contracted postnatally or perinatally. In Indonesia, 4.6% of the population was positive for HBsAg in 1994 and of these, 21% were positive for HBeAg and 73% for anti-HBe; 44% and 45% of Indonesian patients with cirrhosis and HCC, respectively, were HBsAg positive. There appear to be two types of age-specific HBsAg prevalence in the Philippines, suggesting different modes of transmission. In Thailand, 8-10% of males and 6-8% of females are HBsAg positive, with HBsAg also found in 30% of patients with cirrhosis and 50-75% of those with HCC. 

In Taiwan, 75-80% of patients with chronic liver disease are HBsAg positive, and HBsAg is found in 34% and 72% of patients with cirrhosis and HCC, respectively. 

**Figure 3:**
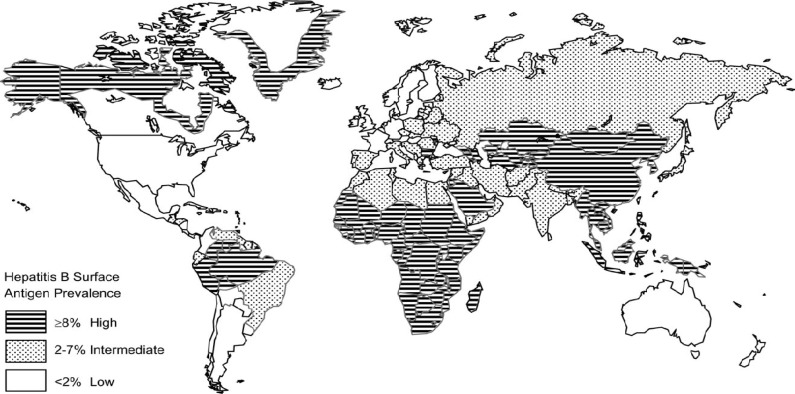
Geographic distribution of the prevalence of chronic hepatitis B virus infection [58].

In China, 73% of patients with chronic hepatitis and 78% and 71% of those with cirrhosis and HCC, respectively, are HBsAg positive. In Singapore, the prevalence of HBsAg has dropped since the introduction of HBV vaccination and the HBsAgsero prevalence of unvaccinated individuals over 5 years of age is 4.5%. In Malaysia, 5.24% of healthy volunteers were positive for HBsAg in 1997 [35]. (Figure 3).

## Discussion

A series of studies have been carried out to assess the viral, host, and environmental cofactors of EBV-associated nasopharyngeal carcinoma, HBV/HCV-associated hepatocellular carcinoma, and HPV-associated cervical carcinoma. Persistent infection and high viral load are important risk predictors of these virus-caused cancers. The International Agency for Research on Cancer (IARC) comprehensively assessed the human carcinogenicity of biological agents. Seven viruses including Epstein-Barr virus (EBV), hepatitis B virus (HBV), hepatitis C virus (HCV), Kaposi's sarcoma herpes virus (KSHV), human immunodeficiency virus, type-1 (HIV-1), human T cell lymphotrophicvirus, type-1 (HTLV-1), and human papillomavirus (HPV) are classified as Group 1 human carcinogens by IARC [36].However, the relevance of viral infection to human cancer development has often been debated. The emerging information may facilitate the development of new molecular-targeted approaches to prevent and treat virally associated human malignancies [37]. Tumor viruses have provided relatively simple genetic systems, which can be manipulated for understanding the molecular mechanisms of the cellular transformation process. A growing body of information in the tumor virology field provides several prospects for rationally targeted therapies. However, further research is needed to better understand the multiple mechanisms utilized by these viruses in cancer progression in order to develop therapeutic strategies [38].

The induction of cancers incidence by exogenous agents, such as chemical, radiation and especially research on *viruses* is an interesting area of active basic subjects and clinical investigation. Prominent research sounds a high prevalence of HBV marker has been found in patients with leukemia as compared to the general population [39,40].The reactivation of HBV was confirmed in patients with AML or other hematological malignancies in various studies. The prevalence of HBV infection was also higher in patients with leukemia than in patients of non malignant control in this study. Danger of hepatocellular carcinoma has been reported vigorously to be highly increased in individual with chronic HBV defect in countries with high and low rates of HBV infection. Overall, general studies are in an accordance with others findings reported from different countries elsewhere [6,41]. On the other hand, several studies suggest a weak or non-significant association of HBV with myeloid leukemia [42] and malignant lymphoma, including NK/Tcell lymphoma and Hodgkin lymphoma [7,43,44]. Further investigation of into the effect of HBV on myeloproliferative malignancies is required to settle these disagreements.

Cases of acute myeloid leukemia which were more frequently positive for HBV- DNA in bone marrow specimen indicate the susceptibility of hemopoietic to hepatitis B infection. In the current literature, the evaluation bone marrow sample of present data also indicates that HBV is associated with chronic and acute myeloid leukemia, particularly with acute myeloid leukemia.

But one study reported that, association of HBV with other subtypes of leukemia is uncertain [7]. Several cancer types reported among HBV positive carriers in the literatures. The risk of pancreatic cancer among HBV carriers was investigated previously, but the results were inconsistent because of the limited number of cases [48,49].The risk of pancreatic cancer was increased among HBV patients who were infected at a younger age, suggesting possible link among them and need further study to confirm it [27]. A few other cancers, such as endometrial, upper aerodigestive tract (larynx), and thyroid gland tumors were found to be associated with HBV infection, but they were observed in either Swedes or immigrants, which call for further investigation. It was reported that interferon alfa therapy against HBV infection can lead to thyroid dysfunction [50], which may increase the risk of thyroid gland tumors [51]. An increased risk of oral cancer was reported recently among patients with HCV supporting the possible link between chronic hepatitis viral infection and oral cancer [52-54]. Furthermore, cancers at a few other sites have been shown inconsistently to be associated with chronic HBV infection. However, geographic heterogeneity in the pathogenesis of leukemias and HBV must be at the urgent discussion [55,56]. Evidence for associations with other cancer sites/types, such as cholangiocarcinoma liver cancer and non-Hodgkin lymphoma, is considered insufficient by LARC.

## Conclusion

Conclusions from reports examining associations with other cancer sites are hardly acceptable because in counties with high rates of HBV infection, there is no cancer registration or its quality is poor. On the other hand, the incidence of HBV infection in the countries which have good quality cancer registration is low. At the present time, the best strategy for decreasing the incidence ofhepatitis B-virus is prevention infectionthrough vaccination. The findings of other studies as well as peer review papers can be as another piece of evidence in support of HBV infection association with blood disorder. However, further studies with larger population of blood disorder patients are needed for drawing firm conclusion.
